# Electrophysiological fingerprints of OFF bipolar cells in rat retina

**DOI:** 10.1038/srep30259

**Published:** 2016-07-26

**Authors:** Alex H. Vielma, Oliver Schmachtenberg

**Affiliations:** 1 Centro Interdisciplinario de Neurociencia de Valparaíso, Facultad de Ciencias, Universidad de Valparaíso, Valparaíso, Chile

## Abstract

Retinal bipolar cells (BCs) divide photoreceptor output into different channels for the parallel extraction of temporal and chromatic stimulus properties. In rodents, five types of OFF BCs have been differentiated, based on morphological and functional criteria, but their electrophysiological characterization remains incomplete. This study analyzed OFF BCs with the patch clamp technique in acute slices of rat retina. Their specific voltage-dependent currents and glutamate responses are shown to represent individual fingerprints which define the signal processing and filtering properties of each cell type and allow their unequivocal identification. Two additions to the rat BC repertoire are presented: OFF BC-2′, a variation of BC-2 with wider axonal arbours and prominent Na^+^ currents, is described for the first time in rodents, and OFF BC-3b, previously identified in mouse, is electrophysiologically characterized in rat. Moreover, the glutamate responses of rat OFF BCs are shown to be differentially sensitive to AMPA- and kainate-receptor blockers and to modulation by nitric oxide (NO) through a cGMP-dependent mechanism. These results contribute to our understanding of the diversity and function of bipolar cells in mammals.

In the mammalian retina, five classes of neurons provide extensive processing and filtering of raw data input, extracting spatial, temporal and chromatic information from the visual scene. The discovery of parallel processing of visual signals by ON and OFF channels in the retina, responding antagonistically to light stimulus increments or decrements, has been a milestone in vision research^1^,^2^. This division of visual input is achieved at the first retinal synapse, formed by photoreceptors, horizontal and bipolar cells (BCs). While ON BCs respond to glutamate liberation from photoreceptors with sign-inverting membrane hyperpolarization mediated by mGluR6 receptors coupled to TRPM1 channels^3^,^4^, OFF BCs express ionotropic glutamate receptors at this synapse, generating sign-conserving depolarizing cationic currents in response to glutamate^5^,^6^,^7^.

Within OFF BCs, the information is shaped by differential contributions of voltage-dependent Na^+^, Ca^2+ ^8,9 and K^+^ conductances^10^, including hyperpolarization-activated cyclic nucleotide-gated (HCN) channels^11^. Inhibitory signalling from different types of amacrine cells, mediated by GABA and glycine receptors^12^, as well as retinal neuromodulators like acetylcholine (ACh) and nitric oxide (NO) contribute to the conditioning of BC responses^13^,^14^. Finally, the processed information is passed on via glutamate release to ganglion cells in the outer half of the inner plexiform layer (IPL).

A large body of evidence, accumulated during the last two decades, has revealed significant differences among mammals regarding the number and relative percentage of OFF BC types, their morphology and glutamate receptor subunit composition at the photoreceptor synapse^15^,^16^,^17^,^18^. In mouse, the five established types of OFF BCs are labelled 1, 2, 3a, 3b and 4, and their axonal arbours stratify in sublayers 1 and 2 of the IPL^19^,^20^, comprising about 40% width of this synaptic stratum^21^,^22^. Although important progress in the functional differentiation of OFF BCs in different species, including mouse, ground squirrel and rat, has been achieved during recent years^15^,^16^, the organizational scheme of BCs is still mostly based on morphological criteria, particularly axonal arbour shape and localization with respect to the IPL sublayers. Physiological studies of BC function are hindered by the fact that these cells cannot be reliably identified in retinal whole mounts and slice preparations without dye filling or immunohistochemical processing, and transgenic mouse lines expressing fluorescent markers in specific BC types, although becoming increasingly popular, may not always be available or experimentally suitable. Here, we present an electrophysiological approach to unequivocally distinguish OFF BCs in rat retina, based on their voltage-gated currents and responses to glutamate stimuli under voltage and current clamp. This study expands the number of known OFF BC types from 4^6^,^11^,^18^ to 5 in rat, as in ground squirrel^23^ and mouse^15^,^21^, and presents a variation of BC-2 expressing prominent Na^+^ currents. Moreover, our results show that glutamate responses in BC-2, 3b and 4 depend on both AMPA and kainate receptor activation and are subject to inhibitory modulation by NO.

## Results

The present study was designed to provide a comprehensive analysis of both the morphological and electrophysiological characteristics of rat OFF BCs. A total of 1190 retinal BCs were recorded with the patch clamp technique. 415 of these, corresponding to 35%, were identified as OFF BCs in accordance with axon stratification in the OFF sublayer of the IPL. OFF BC types were initially defined based on established morphological and electrophysiological criteria for rat and mouse retina^6^,^15^. BC-1 and BC-2 were differentiated according to their axonal and dendritic arbour width, while the identity of BC-3a, 3b and 4 was supported by immunohistochemistry against HCN4, PKARIIβ and calsenilin. In cases of morphological ambiguity, the voltage-gated current pattern was taken into consideration. In total, 6 cells were classified as BC-1 (1.4%), 33 as BC-2 (8%), 8 as BC-2′ (1.9%), 124 as BC-3a (29.9%), 92 as BC-3b (22.2%) and 152 as BC-4 (36.6%).

### Specific properties of OFF BC types

#### BC-1

**Figure 1:**
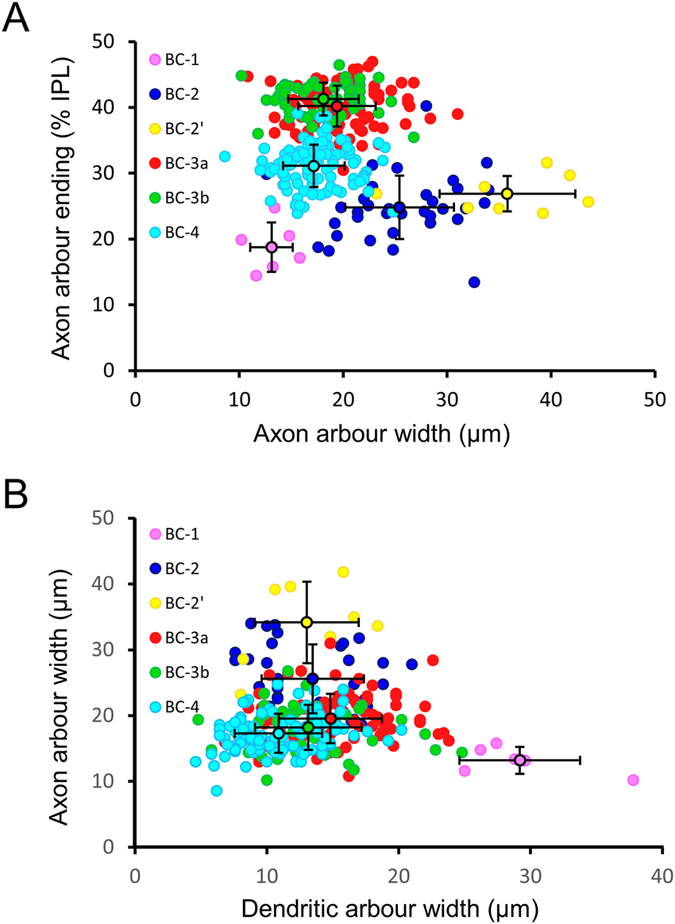
Morphological differentiation of OFF BC groups. (**A**) The distribution of BC axonal arbour ending depth in the IPL versus its horizontal extension displays a separation of all OFF BC groups, except for BC-3a and 3b. Notably, the classification of BC-2′ as a separate group based on electrophysiological criteria, is supported by its wider axonal arbour compared to BC-2. (**B**) Plotting axon arbour versus dendritic arbour width clearly distinguishes BC-1 from BC-2 and the other BC cell types. Black circles and bars indicate the average ± s.d.

**Figure 2:**
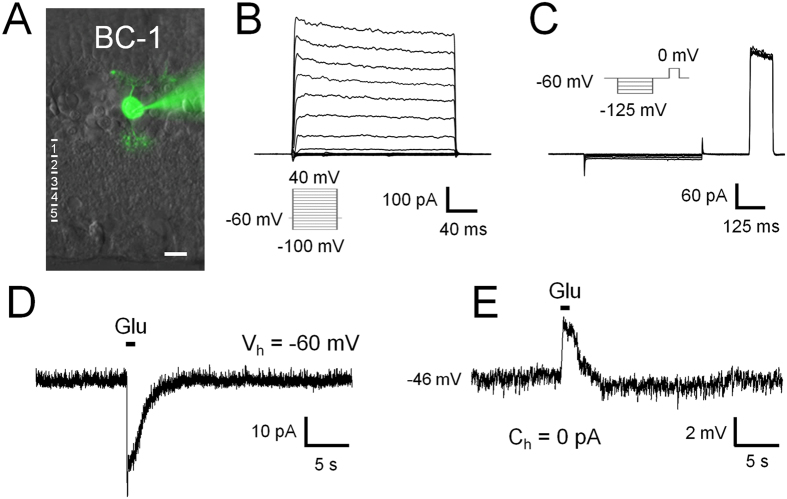
Electrophysiological characteristics of BC-1. BC-1 (**A**) responded to hyper- and depolarizing voltage steps only with outward currents. Neither characteristic transient nor sustained inward currents were observed (**B**,**C**). In response to an L-glutamate stimulus (Glu; 1 s duration), inward currents developed under voltage clamp at −60 mV and deactivated within 5 s (**D**). Under zero-current clamp, glutamate triggered only small depolarizations with a similar time course (**E**). The numbers in (**A**) indicate the IPL sublayers. The insets in (**B**,**C**) indicate the voltage step protocol. Scale bar = 10 μm in this and the following images.

This cell type was the most rarely encountered OFF-BC in our and previous electrophysiological studies in rat^6^,^11^. The axons of BC-1 ramify in the outermost sublayer of the IPL, while their cell bodies are located to the centre of the INL (Figs 1 and 2A). A BC was classified as BC-1 if its dendritic arbour was wider than 20 μm and its axonal arbour, stratifying in sublamina 1, smaller than 20 μm (Fig. 1 and Table 1). Indeed, the dendritic arbours of BC-1 are significantly wider than those of all other OFF BCs in rat (p < 0.05; ANOVA followed by Bonferroni’s Multiple Comparison Test; Fig. 1 and Table 1). The voltage-gated currents of BC-1 consist of outward currents without significant inward currents in our recording conditions (Fig. 2B,C). Glutamate stimulation under voltage-clamp at −60 mV triggered a transient inward current (18 ± 10 pA, n = 3) of long time-to-peak (546 ± 93 ms, n = 3; Fig. 2D), while the glutamate response under zero-current clamp consisted of a small depolarization with a similar time course (Fig. 2E).

#### BC-2

**Figure 3:**
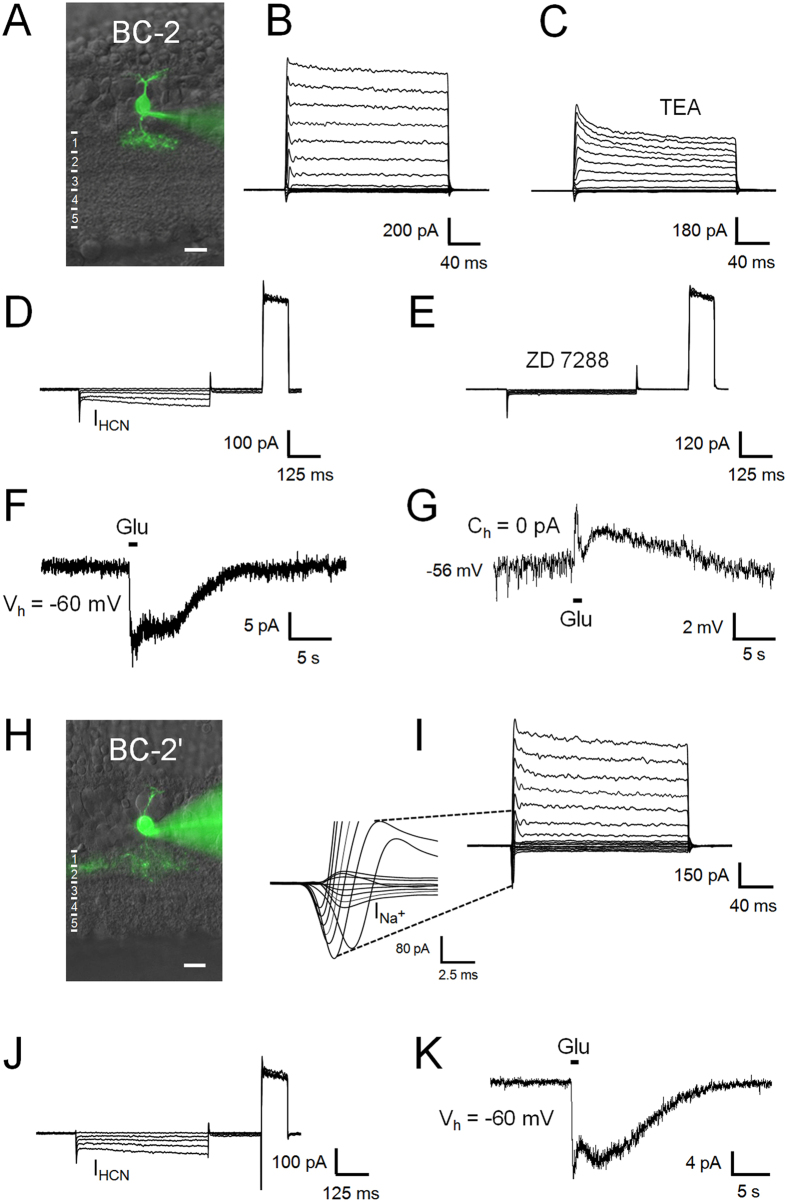
Electrophysiological characteristics of BC-2 and 2′. BC-2, characterized by a wider axonal than dendritic arbour (**A**), generated outward currents sensitive to TEA (**B**,**C**) and inward HCN currents (I_HCN_; **D**) sensitive to ZD 7288 (**E**). BC-2 responded to glutamate with comparatively small, bi-phasic inward currents (**F**). Accordingly, under zero-current clamp, glutamate caused an initial transient and a secondary, slow depolarization (**G**). The group of BC-2′ (**H**) expressed comparatively smaller outward and prominent sodium currents (I_Na_+) (**I**). HCN-currents and glutamate responses were similar between the two cell types (**J,K**).

The cell bodies of BC-2 are generally localized to the centre or the inner half of the INL, and the axonal arbour spreads across sublayer 1 of the IPL (Fig. 3A). However, the axonal arbour is significantly wider than that of BC-1, while its dendritic branches expand significantly less in comparison (p < 0.05 for both; ANOVA followed by Bonferroni’s Multiple Comparison Test; Fig. 1 and Table 1). The maximum outward current, measured at V_h_ = 40 mV, was 875 ± 51 pA (n = 28) and sensitive to 10 mM TEA (Fig. 3B,C). As opposed to BC-1, these cells present I_h_ currents (Fig. 3D)^11^, that were blocked with ZD 7288 (Fig. 3E). The glutamate response was of small amplitude (12 ± 2 pA, n = 14) and long time-to-peak, similar to BC-1 (479 ± 48 ms, n = 14; Fig. 3F). A separation of the glutamate response into a transient and a sustained component^6^,^13^ is evident under zero-current clamp in BC-2 (Fig. 3G).

#### BC-2′

We encountered a variation of BC-2, here denominated BC-2′, that presents an overall morphology and dendritic arbour width similar to BC-2 (Fig. 3H), although its axonal tree diameters were significantly larger (p < 0.05; ANOVA followed by Bonferroni’s Multiple Comparison Test; Fig. 1 and Table 1). In addition, this cell type revealed significant and consistent variations in its voltage-gated currents compared to BC-2. The maximum outward current was of significantly smaller amplitude in BC-2′ than in BC-2 (553 ± 61 pA, n = 8, p = 0.027, t-test for unpaired samples), and conspicuous TTX-sensitive Na^+^ currents developed upon depolarization (214 ± 35 pA, n = 8; Supplementary Fig. S1), characterized by fast activation (2.2 ± 0.5 ms time-to-peak) and inactivation, which are absent from BC-2 (Fig. 3I). I_h_ currents (Fig. 3J) and glutamate responses (Fig. 3K) were of similar kinetics as in BC-2. The latter were of comparatively small amplitude (14 ± 4 pA, n = 5) and long time-to-peak after the stimulus onset (474 ± 76 ms, n = 5), and the average transferred charge in response to glutamate was higher than in BC-2 (Table 1).

#### BC-3a

**Figure 4:**
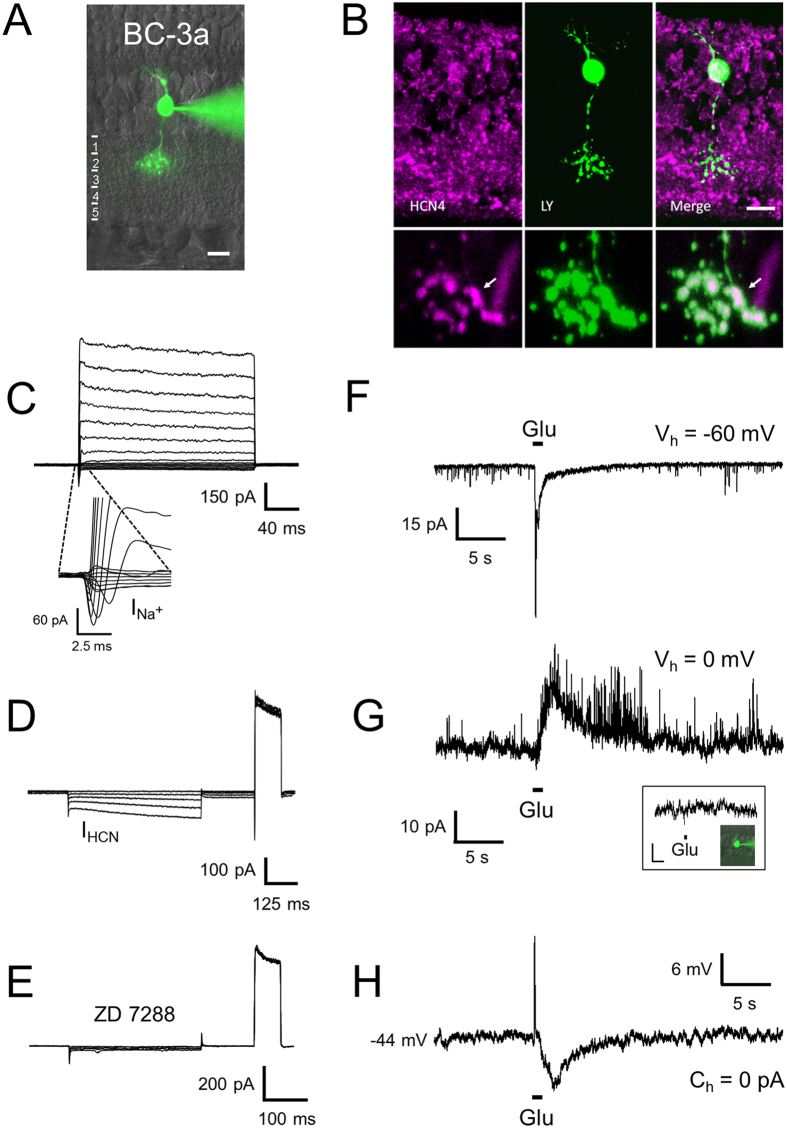
Electrophysiological characteristics of BC-3a. The axonal arbour of BC-3a stratifies across the second sublayer of the IPL (**A**). Its identity was further confirmed by HCN4 immunohistochemistry of previously recorded cells (**B**, arrows). Note that the higher magnification images are from a different cell. Upon depolarization, BC-3a expressed outward currents of similar amplitude as BC-2′, but smaller Na^+^ currents (**C**), while hyperpolarizing voltage steps activated HCN currents (**D**), sensitive to ZD 7288 (**E**). Glutamate stimuli triggered fast transient inward currents without a discernible secondary, sustained component (**F**). Voltage-clamped to 0 mV, glutamate stimulation evoked inhibitory responses (**G**) except in axotomized cells (inset; bars: 4 pA, 5s), which caused prolonged hyperpolarization under zero-current clamp, after an initial fast depolarization (**H**).

This cell type corresponds to the hitherto denominated type 3 OFF BC in rat retina^18^,^24^. Due to its matching morphological and immunocytochemical characteristics, we consider it homologous to mouse BC type 3a^15^. Its cell body typically localizes to the centre of the INL, and its axonal arbour spreads across sublayer 2 of the IPL (Fig. 4A). The tentative identification of BC-3a was confirmed by immunohistochemical labelling of previously electrophysiologically characterized and dye-filled cells for the expression of HCN4 channels^24^,^25^ (n = 7), which has been established as a specific marker for BC-3a in mice^26^ (Fig. 4B).

The pattern of voltage-gated currents of BC-3a is similar to BC-2′, but its HCN currents are of larger amplitude (82 ± 2 versus 60 ± 11 pA at −125 mV, n = 33; p = 0.014, t-test for unpaired samples; Fig. 4C,D). The identity of these currents was confirmed by their blockage with ZD 7288^27^ (Fig. 4E). BC-3a typically also produces small-amplitude TTX-sensitive Na^+^ currents (115 ± 14 pA, n = 18), as described for the formerly denominated BC type 3 in rat^9^ (Fig. 4C and Supplementary Fig. S1). However, Na^+^ currents were observed in only 64% of all recorded type 3a BCs (n = 79 of 124 cells), as reported previously in that study. The glutamate response of BC-3a consists of a rapidly activating and inactivating monophasic component with an amplitude of 78 ± 5 pA and an average time-to-peak of 115 ± 8 ms (n = 50; Fig. 4F). Inhibitory signalling is evident at 0 mV, the reversal potential of non-specific cationic currents under our recording conditions, in response to glutamate stimuli (Fig. 4G). These inhibitory currents were absent from axotomized cells, supporting feedback via amacrine cells in the IPL (Fig. 4G, inset; n = 3) and could be blocked completely by coapplication of GABA and glycine receptor antagonists (Supplementary Fig. S2). Under zero-current clamp, direct glutamate stimulation and inhibitory feedback to this stimulus translate into a fast depolarization followed by a prolonged, slow hyperpolarization with a time-course similar to the inhibitory currents under voltage clamp (Fig. 4H).

#### BC-3b

**Figure 5:**
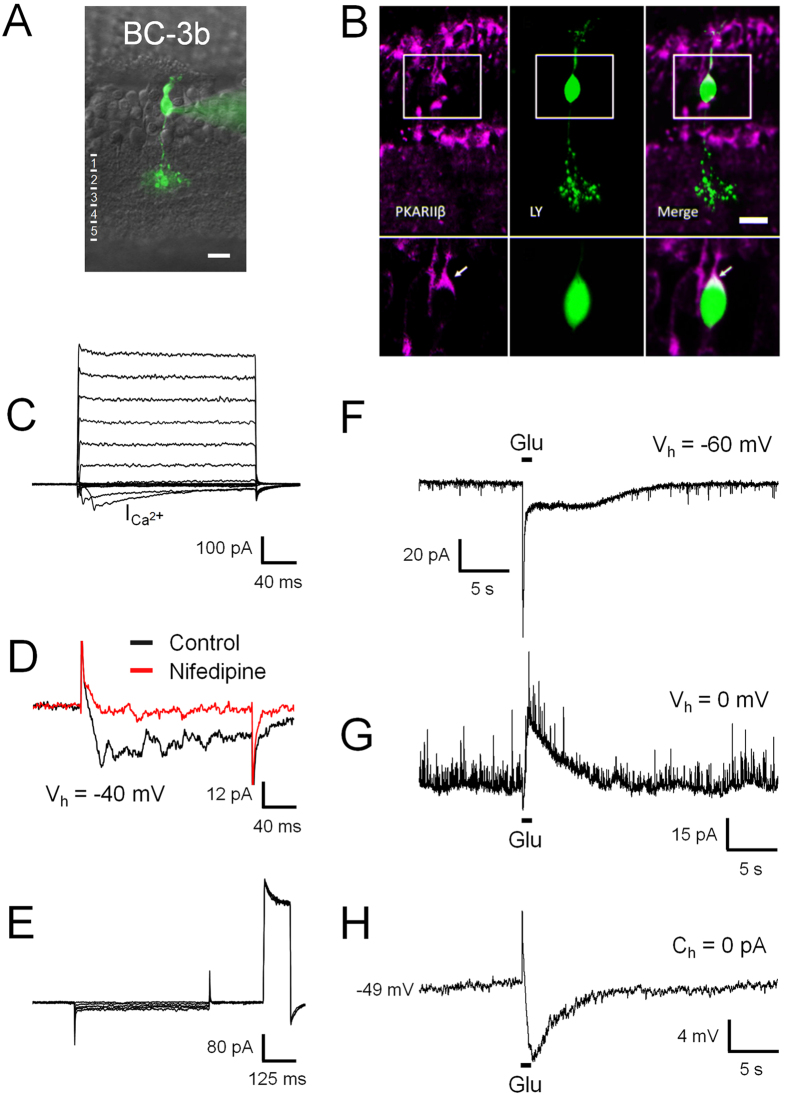
Electrophysiological characteristics of BC-3b. BC-3b is morphologically indistinguishable from type 3a (**A**), but it can be differentiated from the latter by the expression of the RIIβ-subunit of PKA (**B**, arrows). Furthermore, BC-3b displayed slowly inactivating L-type Ca^2+^ currents sensitive to nifedipine (**C**,**D**), but lacked evident HCN currents (**E**). In response to glutamate, BC-3b generated a bi-phasic response consisting of a transient current peak followed by a sustained component of smaller amplitude (**F**). Inhibitory feedback to glutamate stimulation was evident at 0 mV (**G**), generating a prolonged hyperpolarization under zero-current clamp after an initial depolarizing response peak (**H**).

An OFF BC with characteristics similar to mouse BC-3b^15^ was frequently observed in rat retina. The morphological profile of this OFF BC type is similar to type 3a, with a cell body in the centre of the INL and axonal ramification confined to the second sublayer of the IPL (Fig. 5A). Dendritic and axonal arbour extensions were indistinguishable from BC-3a (ANOVA followed by Bonferroni’s Multiple Comparison Test; Fig. 1 and Table 1). To confirm the existence of a homologue to mouse BC-3b in rat, previously recorded and dye-filled candidate cells were immunolabelled for the regulatory subunit RII of protein kinase A (PKARIIβ; n = 4), which has been shown to be a specific marker for this cell type in mouse, serving to differentiate BC-3b from type 3a^26^. Cytoplasmic expression of PKARIIβ was evident in putative BC-3b cells, confirming their homology to this cell type in mouse (Fig. 5B). No colocalization was observed in double immunohistochemical labelling of HCN4 and PKARIIβ, which confirms that BC-3b corresponds to a separate population of BC-3 cells in rat, as in mouse (Supplementary Fig. S3). BC-3b displays neither Na^+^ nor HCN currents under our experimental conditions; instead its voltage step-elicited currents depict characteristic inward currents between −40 and −30 mV with slow activation times (28 ± 2 ms; n = 28) compared to Na^+^ currents (Fig. 5C). These currents were sensitive to the L-type Ca^2+^ channel blocker nifedipine (Fig. 5D) and are therefore most likely L-type Ca^2+^ currents, similar to those encountered in mouse BC-3b^8^. The glutamate response of BC-3b displays a transient component as in BC-3a, with an average amplitude of 83 ± 10 pA and a time-to-peak of 91 ± 7 ms, and a long-lasting sustained component of smaller amplitude (7 ± 1 pA, n = 27; Fig. 5F). This response to glutamate and the inhibitory feedback from other retinal neurons (Fig. 5G) generate a biphasic response under zero-current clamp: An initial fast depolarization followed by a prolonged hyperpolarization, similar to that observed in BC-3a (Fig. 5H).

#### BC-4

**Figure 6:**
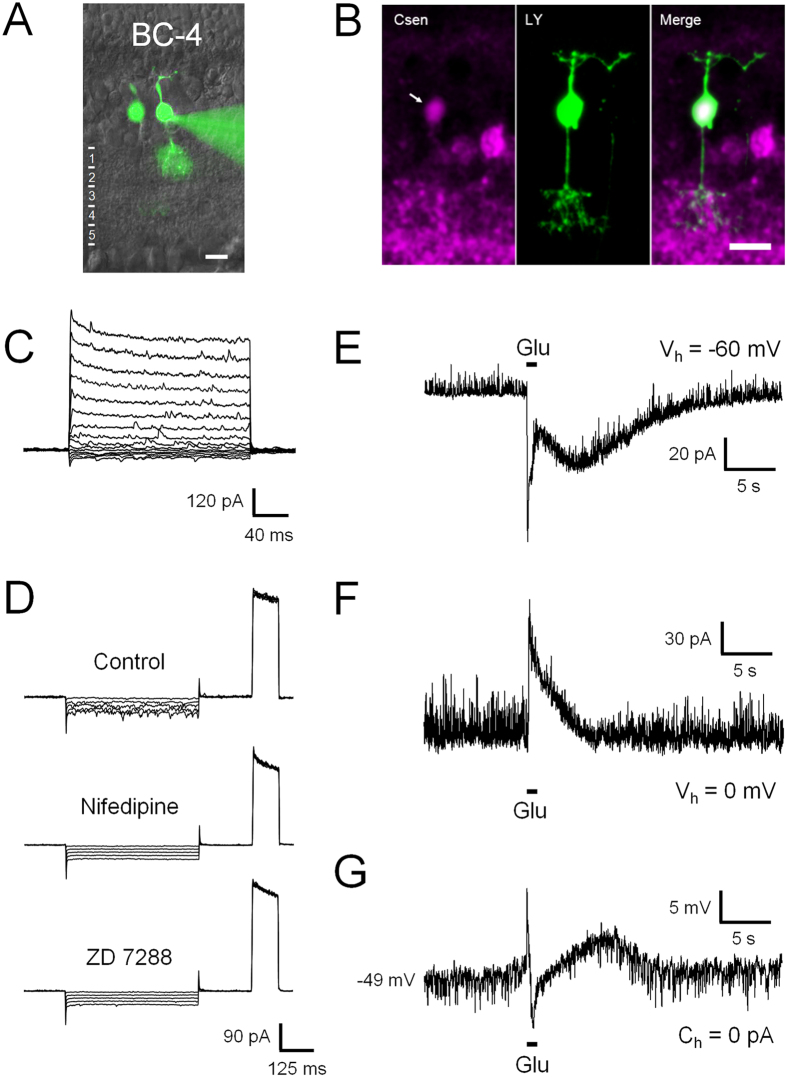
Electrophysiological characteristics of BC-4. BC-4 is morphologically characterized by the spread of its axonal arbour across the sublayers 1 and 2 of the IPL (**A**). After recording, its identity was immunohistochemically confirmed by calsenilin (Csen) labelling (**B**, arrow). Increasingly depolarizing voltage steps caused first inward and subsequently outward currents with a large amount of synaptic noise, but no evident Na^+^ currents (**C**). Hyperpolarization triggered non-inactivating inward currents insensitive to both nifedipine and ZD 7288, suggesting the presence of I_Kir_ currents (**D**). In response to glutamate, a fast initial response is followed by a slowly developing secondary component under voltage clamp (**E**). Together with inhibitory feedback (**F**), this generates a complex triphasic response under current clamp (**G**).

The cell bodies of BC-4, the most frequently recorded OFF BC of the present study, are located to the centre or inner part of the INL, and their axonal arbours spread across the two OFF-sublaminae of the IPL (Fig. 6A). Interestingly, the axonal arbour of BC-4 does not extend across the entire OFF-sublamina as in mouse^15^ (Fig. 1 and Table 1). The identity of this BC type was confirmed by the expression of calsenilin (Fig. 6B), a marker of type 4 BCs in mouse (Supplementary Fig. S3)^28^. The voltage-gated current pattern of BC-4 consists mainly of outward currents, superimposed on which a high degree of spontaneous, inhibitory post-synaptic current activity can be observed (Fig. 6C). This activity is likely to be glycinergic, as it was completely blocked by strychnine (Supplementary Fig. S2). In addition, BC-4 produces inward I_h_ currents resistant to both nifedipine and ZD 7288, suggesting that they are inwardly rectifying K^+^ (I_Kir_) currents^10^ (Fig. 6D). BC-4 presents a complex response to glutamate, consisting of a transient component with an average amplitude of 82 ± 5 pA and a time-to-peak of 108 ± 8 ms, followed by a large and long-lasting rebound current with an average maximum amplitude of 31 ± 2 pA (n = 46; Fig. 6E). Accordingly, the glutamate response under current clamp displays fast initial depolarization, followed by a transient hyperpolarization due to inhibitory feedback (Fig. 6F and Supplementary Fig. S2), and a final prolonged depolarization of smaller amplitude (Fig. 6G).

### Differential expression of AMPA and kainate receptors in OFF BCs

**Figure 7:**
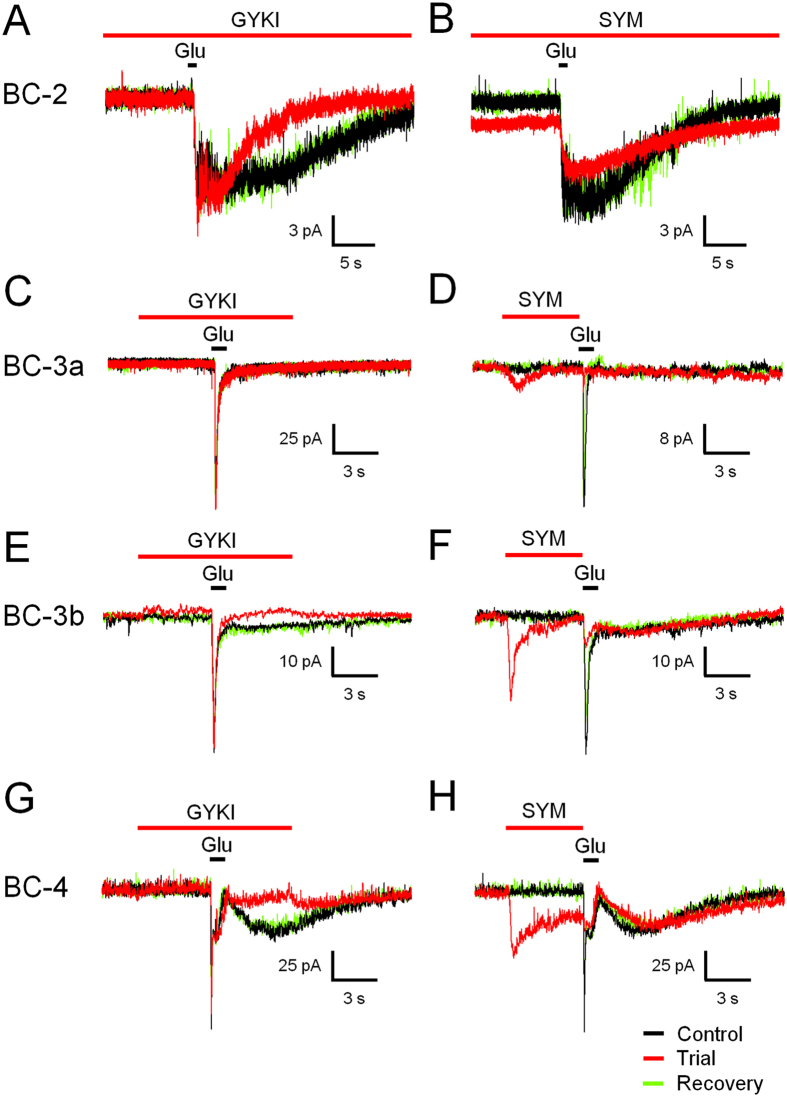
Differential sensitivity of OFF BC types to AMPA- and kainate receptor blockers. GYKI 52466 partly blocked the sustained component of the glutamate response in BC-2, 3b and 4 without affecting the maximum response amplitude (**A**,**E**,**G**), but had no significant effect on BC-3a (**C**). SYM 2081, a desensitizing agonist of kainate-type receptors, partly blocked the responses of all OFF BC types tested (**B**,**D**,**F**,**H**). Note the baseline shift and the transient response caused by SYM prior to the glutamate stimulus in BC-2 and 3b (**B**,**F**). Complete recovery from receptor blockage was observed in all experiments (green traces). Data for BC-1 and 2′ were not included (n = 1) and data for BC-4 have in part been published previously^13^.

The temporal response properties of OFF BCs have been attributed to differential expression of ionotropic glutamate receptor subunits in their dendritic arbours^5^, and a general scheme for mammalian retinas is slowly emerging^7^,^16^,^23^,^29^,^30^. We investigated this problem in rat OFF BCs by application of the AMPA receptor antagonist GYKI, and the kainate receptor antagonist and desensitizing agonist UBP and SYM respectively, the latter of which caused inward currents by itself. Our results show blockage of AMPA receptors in BC-2, 3b and 4 (Fig. 7A,E,G), while kainate receptors were blocked in BC-2, 3a, 3b and 4 (Fig. 7B,D,F,H). Accordingly, the reported exclusive operation of kainate receptors in mouse BC-3a dendrites^7^,^16^ appears to be mirrored in rat, while all other tested BC types were to variable degrees sensitive to both AMPA and kainate receptor blockers (Table 1). However, AMPA receptors dominated the glutamate response in BC-2, while kainate receptors were predominant in BC-3b. Interestingly, application of GYKI tended to shorten glutamate responses without reducing the maximum amplitude, while UBP and SYM reduced the maximum response amplitude, generally without affecting the sustained response component, except for BC-2, in which such a distinction could not be made, and the response was sensitive to both GYKI and SYM (Fig. 7A,B).

### NO modulates the response to glutamate in BC-2 and 3b

**Figure 8:**
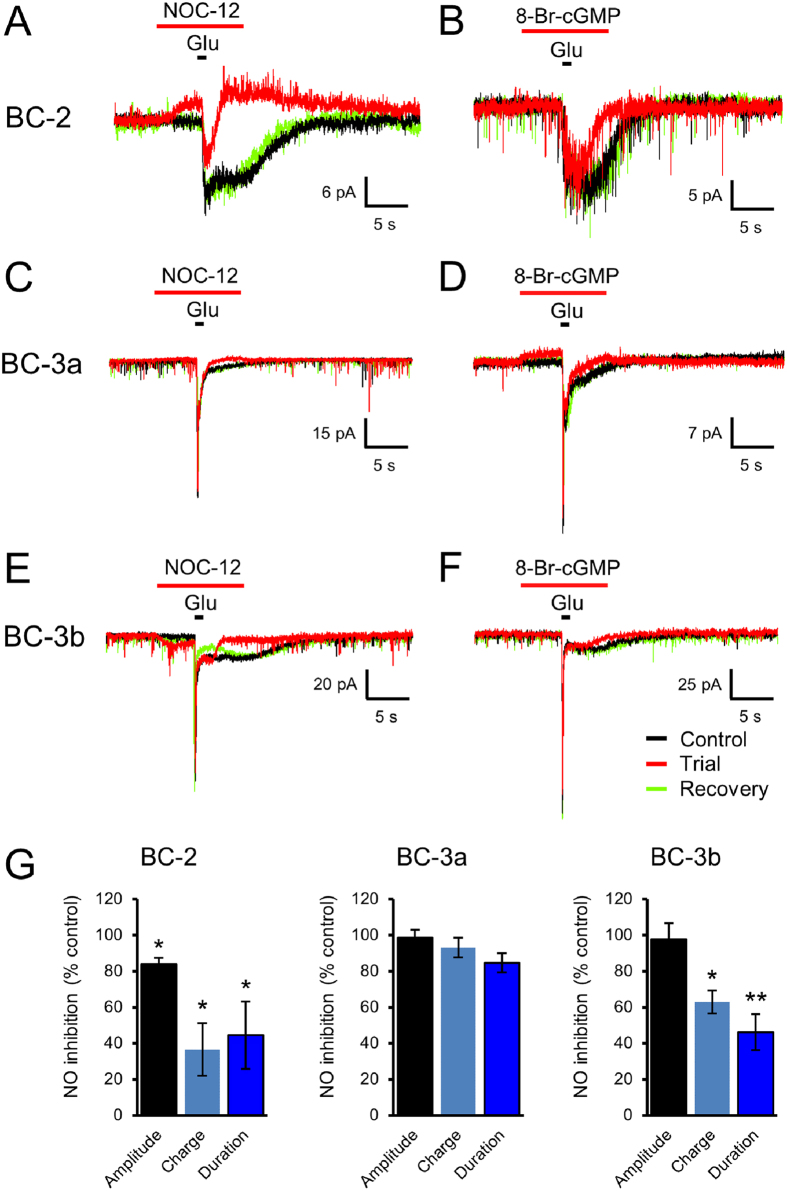
NO and its second messenger cGMP modulate the response to glutamate differentially across OFF BC types. In BC-2, the response to glutamate was significantly shortened by co-application of the NO donor NOC-12. Note that NO also caused a reversible baseline shift by itself (**A**). Application of the membrane-permeable cGMP analogue 8-Br-cGMP shortened the glutamate response similar to NO (**B**). The fast and transient response of BC-3a was unaffected by NO and 8-Br-cGMP (**C**,**D**). In BC-3b, the slow component of the glutamate response was drastically shortened by NO, while the initial fast component was unaffected (**E**). 8-Br-cGMP had a similar inhibitory effect limited to the sustained component of the glutamate response (**F**). (**G**) Bar graphs depicting the modulation of maximum amplitude, transferred charge and duration of responses to glutamate puffs in BC-2, 3a and 3b by NO. Asterisks indicate significant differences compared to control (paired student’s t-test).

Increasing evidence suggests feedback modulation of OFF BCs by NO from specific amacrine cells, termed NOACs^31^,^32^. In rat BC-4, the glutamate response is subject to inhibitory modulation by NO, significantly shortening its duration and total charge transfer^13^. To test if the glutamate response of other OFF-BC types is also modulated by NO, the NO-donor NOC-12 was superfused on the IPL while glutamate was applied to the dendritic arbours in the OPL. Indeed, NO significantly shortened the duration of the glutamate response in BC types 2 and 3b (Fig. 8A,E,G). A similar inhibitory effect on the glutamate response was elicited by stimulation with 8-Br-cGMP instead of NO in these types of cells (Fig. 8B,F), suggesting the operation of a NO-cGMP pathway modulating the glutamate response, as recently described in detail for BC-4^13^. On the other hand, no significant effect of NO and 8-Br-cGMP on the glutamate response was observed in BC-3a (Fig. 8C,D,G).

## Discussion

Visual information enters the mammalian retina through broadly tuned rod and cone photoreceptors and leaves it for higher visual brain centres through about 20 different types of ganglion cells, dedicated to specific aspects of the visual flow. In between, the family of BCs assumes the task to divide the crude information into over 10 processing channels, depending on the species, to filter and condition the visual information and to provide differential connectivity to amacrine and ganglion cells^17^. Together with their separation into OFF and ON cells^2^,^18^, a differential expression of glutamate receptor types and voltage-gated conductances, complemented by diverse inhibitory and modulatory input channels, serves this purpose across the morphologically and functionally discrete BC types^15^.

The present study sought to distinguish OFF BCs by their voltage-gated currents, responses to glutamate, and the potential modulation of this response by NO. The number of cells for which these data could be obtained varied widely across BC types, from 6 (BC-1) to 152 (BC-4). These numbers do not reflect the actual percentage of OFF BC types, which has been shown to be roughly equal by serial block-face electron microscopy in mouse^21^. Similar unequal distributions of recorded cells have been obtained in previous electrophysiological studies^6^,^11^, but which cellular properties convert a BC type into a favourable patch clamp target remains to be shown.

Our data show that while the outward K^+^ current pattern is similar across all OFF BCs, hyper- and depolarization-activated inward currents are comparatively diverse and allow an initial approximation to BC identity^11^. Whereas BC-1 displays no significant inward currents under our recording conditions, BC-2, 2′ and 3a express HCN channels that generate characteristic slowly-activating I_h_ currents at hyperpolarized potentials. However, BC-3b does not express this conductance; instead these cells show prominent L-type Ca^2+^ currents in voltage-step protocols. Although all BCs express voltage-gated Ca^2+^ channels^33^,^34^ (Supplementary Fig. S4), these inward are usually masked in standard depolarizing voltage-step protocols without elevated extracellular Ca^2+ ^34 and block of K^+^ currents, except for BC-3b (Fig. 5C). Finally, BC-4 are distinguished by inwardly rectifying I_Kir_ channels^10^ generating sustained inward currents in the apparent absence of the aforementioned conductances.

On the other hand, the response to glutamate was different in each BC cell type, except for BC-2′, which was indistinguishable from type 2. Both BC-3a and BC-2′ express transient Na^+^ currents upon depolarization, but their response to glutamate is dissimilar, consisting of a large, fast and transient inward current under voltage-clamp in BC-3a, which depends on the activation of dendritic kainate receptors (Fig. 7C,D and Table 1). As BC-3a, BC-3b and 4 also generate a transient glutamate response component, but the response terminates in a sustained current lasting for several seconds, which is responsible for the bulk of the charge transferred during the response under our experimental conditions. The duration of the glutamate response depends on AMPA-type glutamate receptors in BC-4, as evidenced by its sensitivity to GYKI 52466 and insensitivity to SYM 2081^13^. Considering the tonic release of glutamate from photoreceptor terminals under scotopic conditions, the slow time course of this response suggests its participation in modulatory or adaptational processes such as the regulation of the resting membrane setpoint in OFF BCs, rather than a direct transmission of fast visual stimuli.

The finding that voltage- and glutamate-dependent currents are clearly distinct in BC-3b compared to 3a confirms the separate identity of this BC type, initially identified immunohistochemically in mouse^26^, for the rat. BC-2′, expressing Na^+^ currents and significantly smaller K^+^ currents than BC-2, appears in this regard as an intermediate between BC-2 and 3a. However, there are clear differences in both morphology and glutamate responses between BC-2′ and 3a, therefore we classify this cell as a variant of BC-2. If additional differences to the latter are found by future research and if the BC type is also observed in mouse, it should be relabelled as BC-2b.

Rodent OFF BCs express both AMPA and kainate receptors with variable subunit composition in their dendritic endings^5^,^7^,^23^. It has been suggested that the light responses of OFF BCs depend primarily on kainate receptor activation^29^,^30^, while others reported a differential expression and contribution of AMPA and kainate receptors across OFF BCs^5^,^16^,^23^. In mouse, BC-1 responds mostly via AMPA receptors, while only kainate receptors are expressed and functional in BC-2 and 3a^7^,^16^. BC-4 responds to glutamate through both kainate and AMPA receptors in this species. Our data support the notion that both AMPA and kainate receptors contribute, in variable and probably species-specific proportions, to responses in OFF BCs.

Interestingly, the glutamate response time-to-peak varied widely across the BC types, with increasing cell type number and axonal terminations closer to the OFF/ON border in the IPL clearly correlating with faster responses (Table 1). A short response time-to-peak has been associated with the ability to generate sodium spikes in BCs purportedly forming part of faster signal transmission channels^35^. However, Na^+^ current expression, as observed here, does not exactly follow this scheme, since BC-3b and 4 did not produce these currents, while the newly discovered BC-2′ displayed prominent Na^+^ currents. A recent report confirmed light-induced spiking in mouse BC-3a cells^16^, but a spiking variation of BC-2 was not identified in this species.

Responses with similar sustained currents as in BC-3b and 4 have recently been described in OFF BCs of macaque retina stimulated with glutamate and the GluK1 agonist ATPA, and were considered tail or rebound currents generated by kainate receptor activation^29^. However, ATPA also activates AMPA receptors at the applied concentration^36^, therefore a contribution of AMPA receptors to the glutamate response cannot be excluded. Indeed, AMPA receptor expression has been shown in OFF midget BCs of the marmoset^37^, and rebound currents can result from both kainate and AMPA receptor activation^38^,^39^. It is also possible that differences in glutamate receptor identity and composition across species account for the discrepancies regarding AMPA versus kainate receptor activation in OFF BCs. Clearly, the genesis of the sustained component in glutamate responses of certain OFF BCs remains to be investigated.

We recently described the modulation of the glutamate response in BC-4 by NO^13^. NO is an established modulator of retinal signal processing, but its cellular and molecular actions are only beginning to be unravelled^32^,^40^. The NO receptor soluble guanylate cyclase is widely expressed across the INL and the ON and OFF substrata of the IPL, but is mostly absent from the outer retina^41^. NO, presumably released by nNOS-positive amacrine cells^31^, inhibits and shortens the glutamate response, thereby exerting a temporal modulation of signal transmission in this BC type. In BC-4, the AMPA receptor-dependent rebound current was shown to be inhibited by NO through an intracellular soluble guanylate cyclase – cGMP pathway, while the initial, fast kainate receptor-dependent response was unaffected by NO^13^.

Here, we extend these findings to BC-2 and 3b. Although the glutamate response of these BC types is dissimilar, BC-2 and 3b share with BC-4 the generation of a biphasic response with a sustained component that slowly inactivates over several seconds. As in BC-4, exogenous NO shortened the glutamate response in BC-2 and 3b, without affecting its maximum amplitude. These data suggest that BC-2, 3b and 4 share an AMPA receptor-mediated mechanism that generates slowly inactivating responses to comparatively strong glutamate stimuli which are subject to modulation by a NO-cGMP pathway. On the other hand, BC-3a, which only develops a kainate receptor-dependent transient glutamate response, displayed no sensitivity to NO.

Finally, our data reveal a significant inhibitory input to BCs 3a, 3b and 4 in response to glutamate. The presence of GABA and glycine receptors in BC axon terminals^42^,^43^,^44^ indicates that BCs receive inhibitory input via both GABA and glycine released from amacrine cells within the IPL, although in rodents, the inhibition of OFF BCs is mediated predominantly by glycine^12^,^45^,^46^. Accordingly, it is likely that the outward current observed in our recordings at the approximate reversal potential of non-specific cationic conductances reflects indirect inhibitory feedback from GABAergic and/or glycinergic amacrine cells (Supplementary Fig. S2). This is supported by recordings of axotomized cells in which these currents were not observed. However, we cannot completely rule out inhibitory input through dendritic GABA receptors as a product of direct horizontal cell stimulation by glutamate^47^. On the other hand, the variability in the proportion of GABA and glycine receptors in different OFF BC types^48^,^49^ could explain the distinct influence of inhibition on the membrane potential under zero-current clamp in response to glutamate stimulation.

In summary, the present comparative description of electrophysiological properties of rat OFF BCs confirms that each of the six functionally distinguishable BC types has individual glutamate receptor and ion channel combinations, which confer unique filtering and signal processing properties. Under patch clamp, these results allow the unequivocal identification of OFF BC types without transgenic or immunohistochemical labelling. The sensitivity of glutamate responses to NO displayed by several BC types extends the number of cellular NO targets in the retina and supports the involvement of this unconventional neuromodulator in retinal signal processing.

## Methods

### Animals and retinal slice preparation

Sprague Dawley rats were born and raised in the animal facility of the University of Valparaiso, held at 20–30 °C under a 12 h photoperiod with water and food *ad libitum*. Retinal slices were prepared from 3–4 week-old rats irrespective of sex or weight, by procedures described previously^13^. The experimental protocols were approved by the bioethics committee of the University of Valparaiso and in accordance with the bioethics and biosafety regulation of the Chilean Research Council (CONICYT). Briefly, rats were anesthetized deeply by halothane inhalation and sacrificed by decapitation. Eyes were quickly removed and the retina was carefully separated from the sclera in a chamber with extracellular solution, containing (in mM): 119 NaCl, 23 NaHCO_3_, 1.25 NaH_2_PO_4_, 2.5 KCl, 2.5 CaCl_2_, 1.5 MgSO_4_, 20 glucose and 2 Na^+^ pyruvate, aerated with 95% O_2_ and 5% CO_2_, reaching a pH of 7.4. A piece of central retina was embedded in type VII agarose (Sigma), and cut with a vibratome (Leica VT1000S) to 200 μm thickness. Retinal slices were transferred to the recording chamber, sustained by a U-shaped platinum wire, and superfused with oxygenated extracellular solution (flow rate 1 ml/min) at room temperature (20 °C) under conditions of low photopic background illumination (100 lux).

### Electrophysiology

Retinal slices were visualized with an upright microscope (Olympus BX51WI) equipped with a 40x water-immersion objective, infrared differential interference contrast and a cooled CCD camera for brightfield and fluorescence imaging. Images were captured by NIS-Elements D (Nikon) and processed by Image J software (National Institutes of Health) and Adobe Photoshop CS (Adobe Systems Incorporated). Patch clamp recordings were made from different types of OFF BCs, whose tentative identity was corroborated by comparing the axon terminal stratification within the OFF sublamina of the IPL with established values^15^, after dialysis of Lucifer yellow through the patch pipette. Standard intracellular solution contained (in mM): 125 K^+^ gluconate, 10 KCl, 10 HEPES, 2 EGTA, 2 Na_2_ATP, 2 NaGTP and 1% Lucifer yellow. Intracellular solution to record Ca^2+^ currents contained: 90 Cs-methanesulfonate, 20 TEA-Cl, 10 HEPES, 10 EGTA, 10 Na_2_-phosphocreatine, 2 MgATP, 0.2 NaGTP. The pH was adjusted to 7.4 with KOH or CsOH, respectively. Recording electrodes were fabricated using borosilicate glass capillaries (1.5 mm OD, 0.84 mm ID; WPI) and pulled to resistances between 10–15 MΩ on a Flaming/Brown electrode puller (Sutter P-97). Experiments were only performed if the seal resistance was above 1 GΩ. Series resistance was below 30 MΩ and was left uncompensated. The liquid junction potential was calculated to be 14 mV with the Henderson equation and membrane voltage measurements were corrected offline accordingly. Between experiments, cells were held at a resting membrane voltage of −60 mV. 10 mV voltage steps (200 ms duration) from −100 mV to 40 mV were applied to obtain the voltage-dependent current pattern. Hyperpolarization-activated currents (I_h_) were isolated by voltage steps (500 ms duration) from −65 mV to −125 mV, in −15 mV increments, with a final depolarizing step to 0 mV (100 ms duration). Signals were amplified with an EPC7-plus patch clamp amplifier (HEKA Elektronik), filtered at 3 kHz, digitized and sampled at 10 kHz with an A/D board (National Instruments PCI-6221 or Molecular Devices Digidata 1550). Recording were acquired using custom software written in IGOR PRO (Wavemetrics) or PClamp 10.4 (Molecular Devices).

### Stimulation

L-glutamate (500 μM, Sigma-Aldrich), was applied to the outer plexiform layer (OPL) from a single barrel glass pipette with 1 μm inner tip diameter, using a custom-made computer-controlled picospritzer operating at a pressure of 2–3 psi. Cells were voltage clamped at −60 mV, close to the calculated Cl^−^ equilibrium potential of −63 mV under standard recording conditions, to isolate excitatory, non-specific cationic currents; at 0 mV to isolated inhibitory Cl^−^ currents, or current clamped (0 pA) in whole-cell configuration. The L-type Ca^2+^ channel blocker nifedipine (30 μM, Sigma-Aldrich), the K^+^ channel blocker TEA (10 mM, Merck), the HCN channel blocker ZD 7288 (50 μM, Tocris), the NO donor NOC-12 (200 μM, Calbiochem) and the membrane-permeable cyclic GMP analogue 8-Br-cGMP (1 mM, Sigma-Aldrich) were applied to the IPL from single or triple barrel glass pipettes. Picrotoxin (200 μM, Sigma), strychnine (50 μM, Tocris) and TTX (1 μM, Tocris) were applied by bath perfusion. The AMPA and kainate receptor blockers GYKI 52466 (GYKI, 30 μM, Tocris Bioscience), SYM 2081 (SYM, 50 μM, Tocris) and UBP 310 (UBP, 500 nM, Tocris) were applied by bath perfusion or from glass puffer pipettes directed at the OPL. GYKI and UBP were initially dissolved in DMSO, whereas NOC-12 was dissolved in 1 M NaOH and stored as stock aliquots at −80 °C. All other drugs were dissolved in distilled water as stocks prior to their final dilution in extracellular solution.

### Immunohistochemistry

To confirm the identity of BC-3a, 3b and 4, retinal slices that contained previously recorded and dye-filled cells were fixed for 20 minutes in 4% paraformaldehyde in PBS. The sections were washed twice in PBS and blocked for 1 hour with a solution containing: 1% BSA, 1% horse serum and 0.3% Triton X-100 in PBS, pH 7.4. The primary antibodies, rabbit anti-HCN4 (ab5808, Millipore), mouse anti-PKARIIβ (PS114, BD Bioscience) and mouse anti-CSEN (ab99043, Abcam) were diluted 1:250 in the blocking solution and applied overnight at 4 °C. The sections were washed in PBS and incubated for 1 hour at room temperature with the secondary antibody, donkey anti-rabbit Cy3 or donkey anti-mouse-Cy3 (Jackson ImmunoResearch), diluted 1:800 in PBS. Finally, the slices were imaged with a laser-scanning confocal microscope (Nikon C1plus). Image stacks were captured with EZ-C1 software (Nikon), maximum intensity-projected onto a single plane and post-processed with Photoshop CS (Adobe) to adjust image size, colour, brightness and contrast. Note that antibodies against the neurokinin 3 receptor (NK3R; Abcam ab124025, lot GR87175-2) and synaptotagmin II (Syt2; Abcam ab77507, lot GR62988-3), used to differentiate between BC-1 and 2 in mouse^19^,^20^, did not produce specific labelling of the retina in our preparations.

### Data analysis

In voltage step protocols, the maximum outward current corresponds to the average steady state current during the last 20 milliseconds of the voltage step to 40 mV, whereas the maximum sodium and calcium currents and their current-voltage relationships were measured at their respective maximum amplitude. HCN current amplitudes were measured during the last 20 milliseconds of the voltage step to −125 mV. The glutamate response charge was calculated by integrating the area under the current curve after the stimulus onset, compared to pre-stimulus baseline levels, in Origin 8 Pro software. The glutamate response time-to-peak was determined as the difference between the stimulus command and the response peak time, and the response duration as the time from the stimulus command to return to baseline (±3 s.d. of the baseline noise). Data used for statistical analysis had a normal distribution according to the Shapiro-Wilk test. Results are shown as the mean ± s.e.m., and the statistical significance of the difference between two measures was calculated using the paired or unpaired two-tailed Student’s t-test in Graph Pad InStat software. Dendritic and axonal arbour width was measured in ImageJ software and analyzed with one-way ANOVA followed by Bonferroni´s multiple comparison test, with statistical significance defined as p < 0.05.

## Additional Information

**How to cite this article**: Vielma, A. H. and Schmachtenberg, O. Electrophysiological fingerprints of OFF bipolar cells in rat retina. *Sci. Rep.* **6**, 30259; doi: 10.1038/srep30259 (2016).

## Supplementary Material

Supplementary Information

## Figures and Tables

**Table 1:** Summary of the principal morphological and electrophysiological characteristics that differentiate retinal OFF bipolar cells in rat.

Characteristics	BC-1	BC-2	BC-2′	BC-3a	BC-3b	BC-4
Dendritic arbour width (μm)	29 ± 2 (n = 6)	14 ± 1 (n = 29)	13 ± 2 (n = 7)	15 ± 0.4 (n = 89)	13 ± 1 (n = 48)	11 ± 0.3 (n = 91)
Axon arbour width (μm)	13 ± 1 (n = 6)	26 ± 1 (n = 30)	35 ± 3 (n = 8)	20 ± 0.4 (n = 90)	18 ± 0.5 (n = 53)	17 ± 0.3 (n = 101)
Axon ending depth in IPL (%)	19 ± 2 (n = 6)	24 ± 1 (n = 30)	27 ± 1 (n = 8)	40 ± 0.3 (n = 90)	41 ± 0.3 (n = 53)	31 ± 0.3 (n = 101)
Max outward current (pA)	803 ± 92 (n = 5)	875 ± 51 (n = 28)	553 ± 61 (n = 8)	617 ± 15 (n = 90)	742 ± 19 (n = 76)	778 ± 24 (n = 74)
Max sodium current (pA)	—	—	214 ± 35 (n = 8)	115 ± 14 (n = 18)	—	—
Max calcium current* (pA)	nd	67 ± 12 (n = 3)	nd	20 ± 9 (n = 3)	53 ± 12 (n = 3)	52 ± 18 (n = 4)
Max HCN current (pA)	—	45 ± 4 (n = 16)	60 ± 11 (n = 7)	82 ± 2 (n = 33)	—	—
Glu response charge (pC)	64 ± 19 (n = 3)	99 ± 10 (n = 14)	147 ± 31 (n = 5)	20 ± 2 (n = 50)	71 ± 7 (n = 27)	318 ± 19 (n = 46)
Glu response I_max_ (pA)	18 ± 10 (n = 3)	12 ± 2 (n = 14)	14 ± 4 (n = 5)	78 ± 5 (n = 50)	83 ± 10 (n = 27)	82 ± 5 (n = 46)
Glu response time-to-peak (ms)	546 ± 93 (n = 3)	479 ± 48 (n = 14)	474 ± 76 (n = 5)	115 ± 8 (n = 50)	91 ± 7 (n = 27)	108 ± 8 (n = 46)
GYKI blockage (% charge)	nd	59 ± 14 (n = 3)	nd	2 ± 2 (n = 5)	31 ± 9 (n = 3)	54 ± 8 (n = 5)
SYM blockage (% charge)	nd	30 ± 16 (n = 3)	nd	94 ± 2 (n = 3)	66 ± 14 (n = 3)	47 ± 11 (n = 5)
UBP blockage (% charge)	nd	nd	nd	89 ± 4 (n = 3)	nd	27 ± 15 (n = 5)

Values indicate the mean ± s.e.m. nd, not determined; —, undetectable. *Under blockage of K^+^ currents.
