# Supplementary material for: Electrophysiological fingerprints of OFF bipolar cells in rat retina
Source: Sci Rep. 2016 Jul 26;6:30259. doi: 10.1038/srep30259 (PMC4960551; doi:10.1038/srep30259)
Supplement: Supplementary Information [file srep30259-s1.pdf]

Electrophysiological fingerprints of OFF bipolar cells in rat retina

Authors: Alex H. Vielma and Oliver Schmachtenberg

Supplementary Figures

Na^+-currents

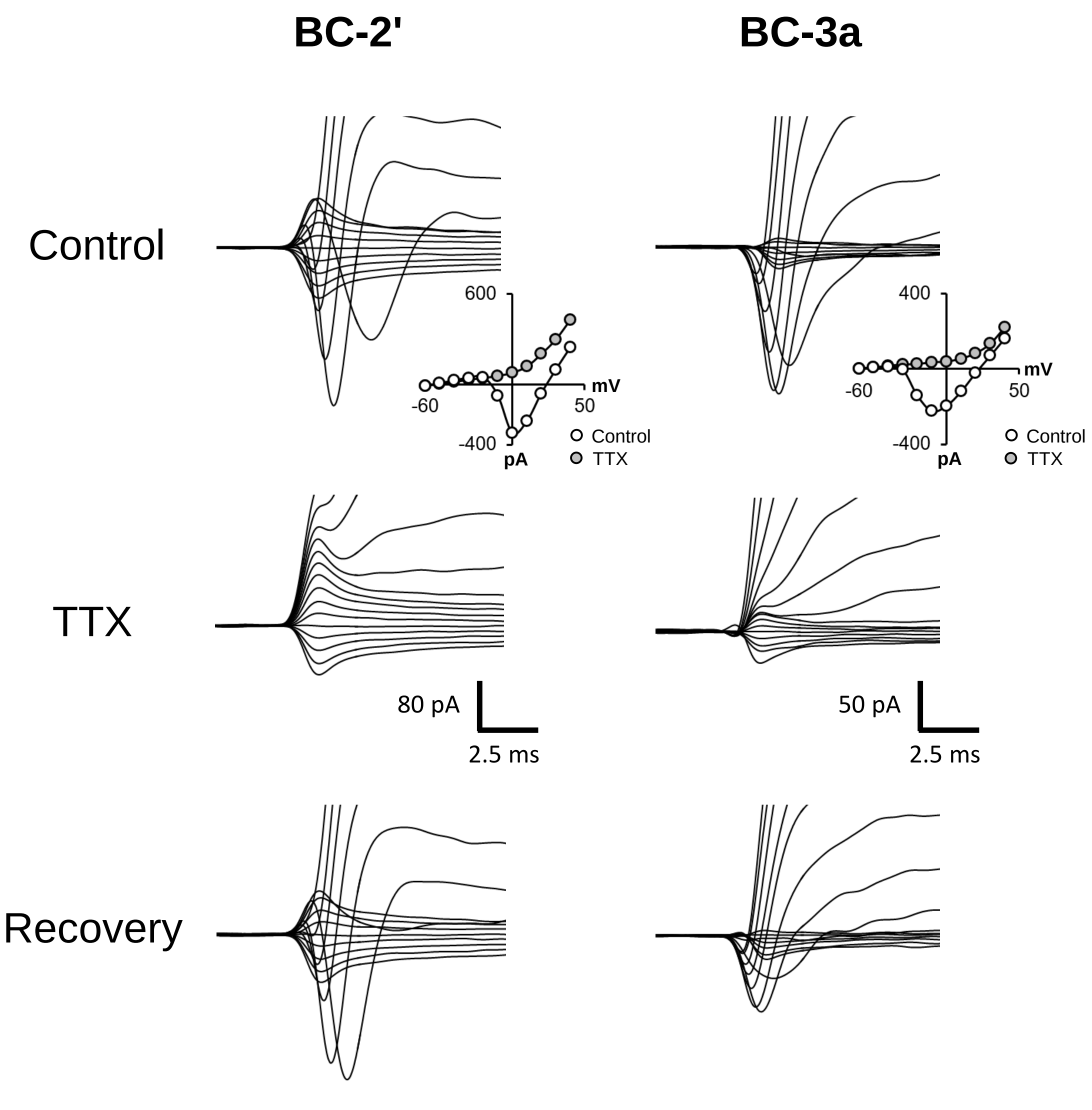

Supplementary Figure S1. Na^+ currents in OFF BC types 2' and 3a. Rapidly inactivating inward currents could be blocked by TTX, confirming their identity as Na^+ currents. The respective I-V relationships are shown below. Note recovery after drug washout.

Inhibitory currents

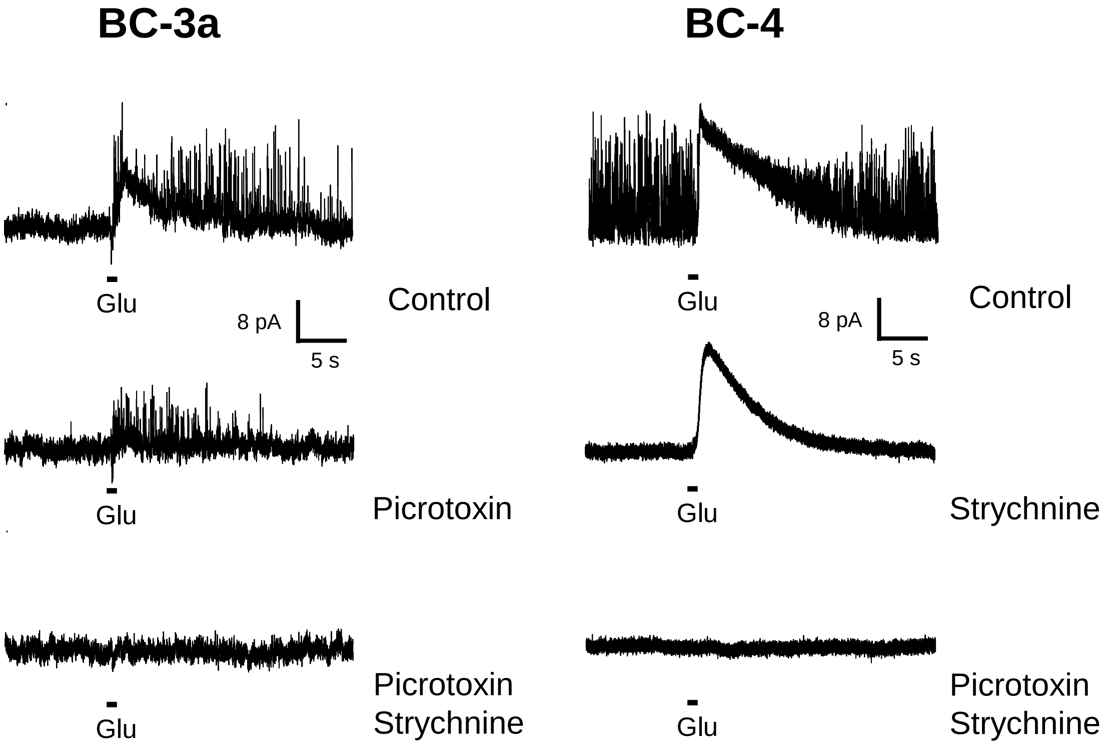

Supplementary Figure S2. Inhibitory currents in OFF BCs. Glutamate-evoked inhibitory currents in BC-3a and BC-4 were sensititve to the GABA receptor blocker picrotoxin and to the glycine receptor blocker strychnine.

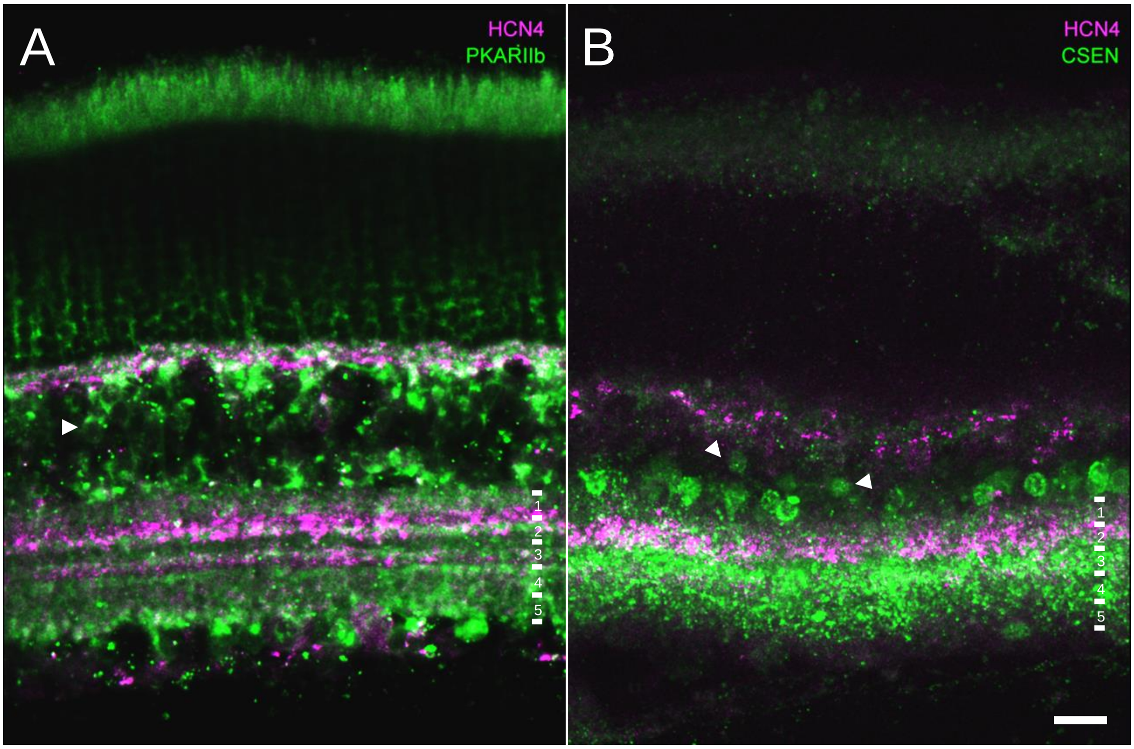

Supplementary Figure S3. Immunohistochemical double labelling with BC markers established for mice [15]. (A) HCN4 expression, a marker of BC-2 cells, revealed a punctate pattern in both the OPL and IPL. Two separate bands in the IPL were evident in some preparations. PKARIIβ immunoreactivity, labelling BC-3b in mice, was present in cell bodies in the outer INL (arrowhead). However, photoreceptors, amacrine and ganglion cells were also partly PKARIIβ-positive. (B) Calsenilin (CSEN) immunoreactivity, which marks BC-4 in mice, was observed throughout the IPL, in amacrine cell bodies and in cell bodies in the outer INL (arrowheads). There was no co-localization of HCN4 with PKARIIβ or CSEN. Scale bar: 20 µm.

Ca^{2+}-currents

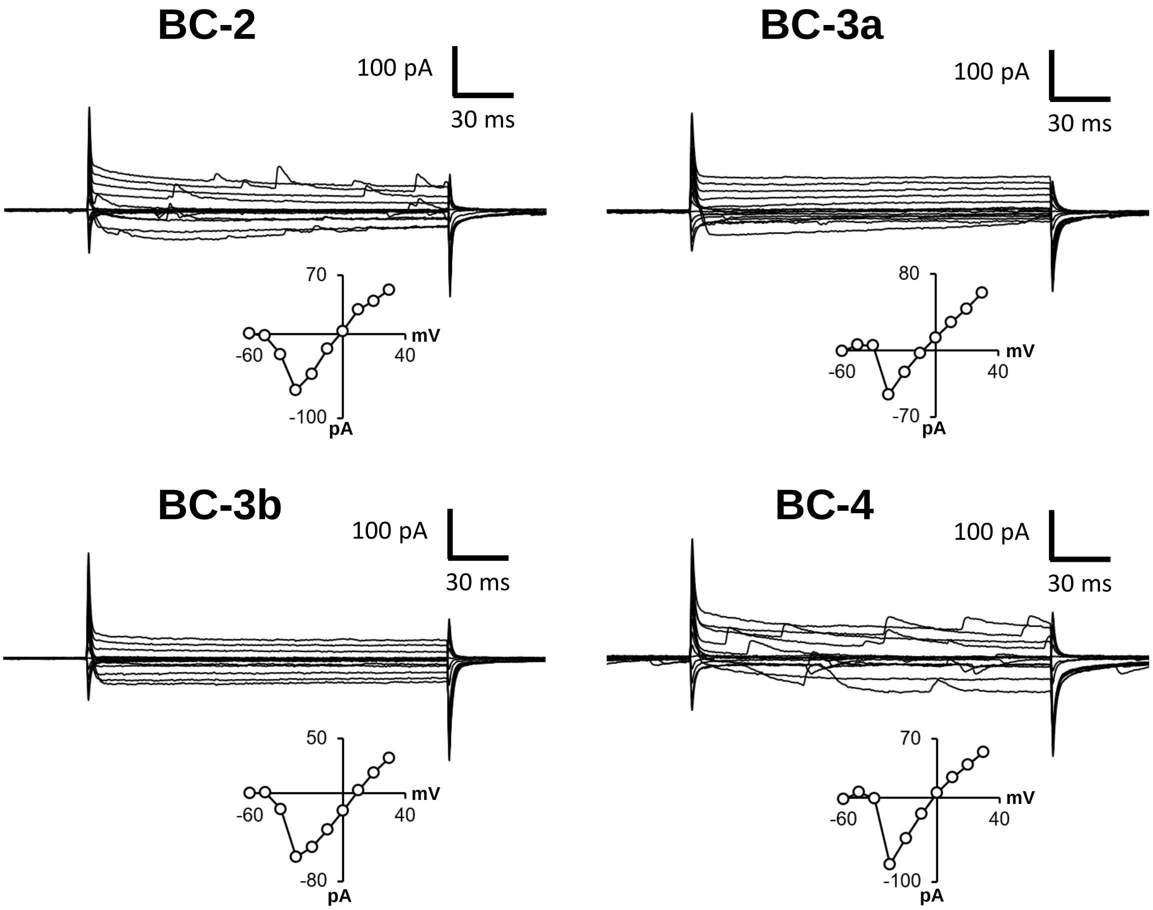

Supplementary Figure S4. Ca^{2+} currents in OFF BCs. Blockage of K^+ currents with Cs^+ and TEA unmasks the presence of Ca^{2+} currents in representative examples of BC-2, 3a, 3b and 4. The respective I-V relationships are shown below.
